# Supplementary material for: OsCRP1, a Ribonucleoprotein Gene, Regulates Chloroplast mRNA Stability That Confers Drought and Cold Tolerance
Source: Int J Mol Sci. 2021 Feb 7;22(4):1673. doi: 10.3390/ijms22041673 (PMC7915912; doi:10.3390/ijms22041673)
Supplement: Supplementary file 1 [file ijms-22-01673-s001.pdf]

Supporting information

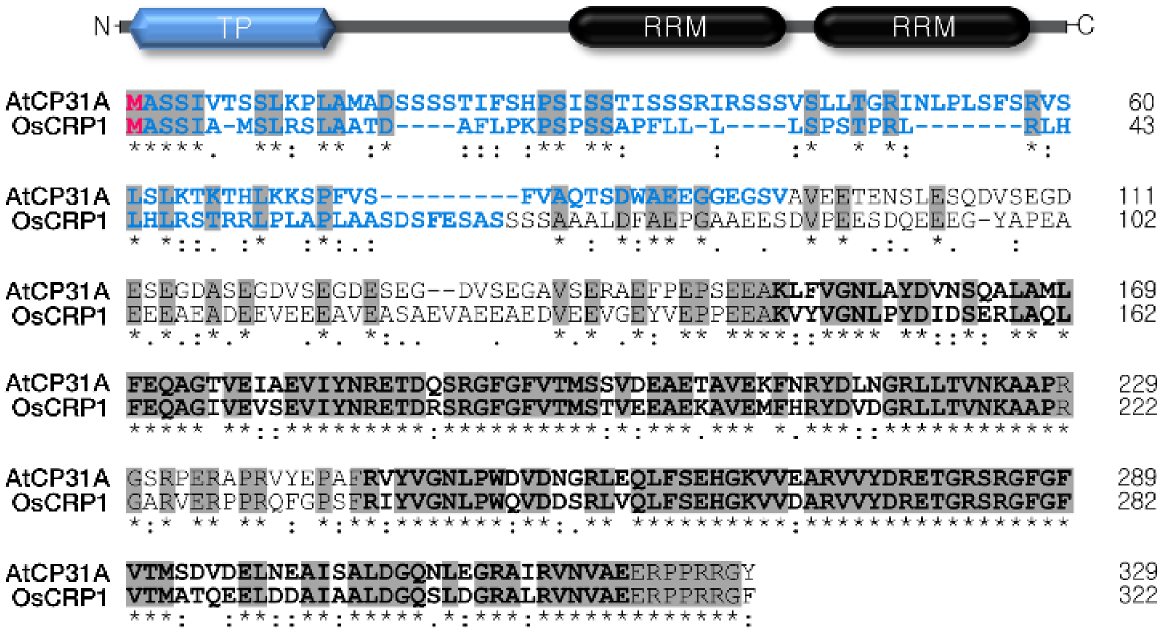

Figure S1. Survey of the conserved motifs of OsCRP1 through alignment with AtCP31A (At4G24770). The alignment was performed with Clustal Omega tool from EMBL-EBI (McWilliam et al. 2013). Blue diamond-shaped box and same color bold letters indicate transit peptide, black ovoid-shaped boxes, and same color bold letters represent RRM (RNA recognition motifs, accession number cd12399) domains. N, amino-terminus, C, carboxyl-terminus, asterisk (*), fully conserved residue that is filled with grey, colon (:), conservation between groups of strongly similar properties (scoring > 0.5 in the Gonnet PAM 250 matrix), period (.), conservation between groups of weakly similar properties (scoring ≤ 0.5).

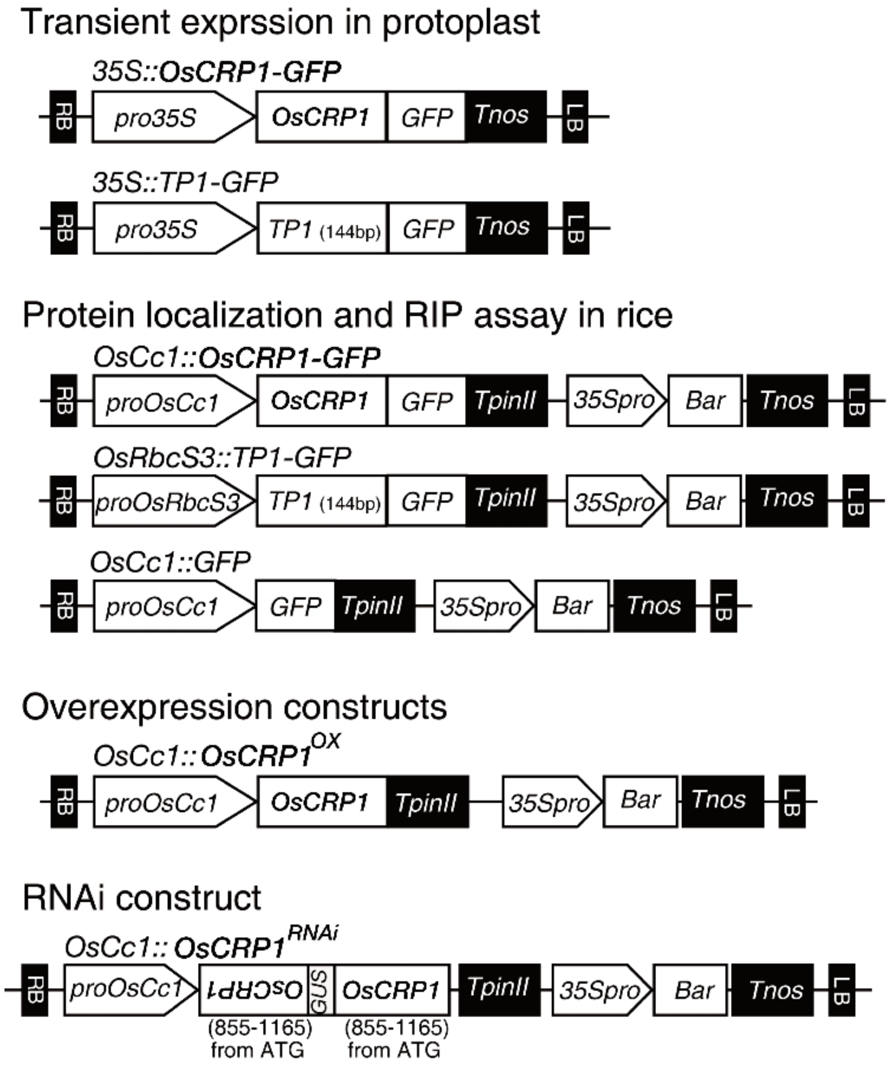

Figure S2. Vectors used in this study.

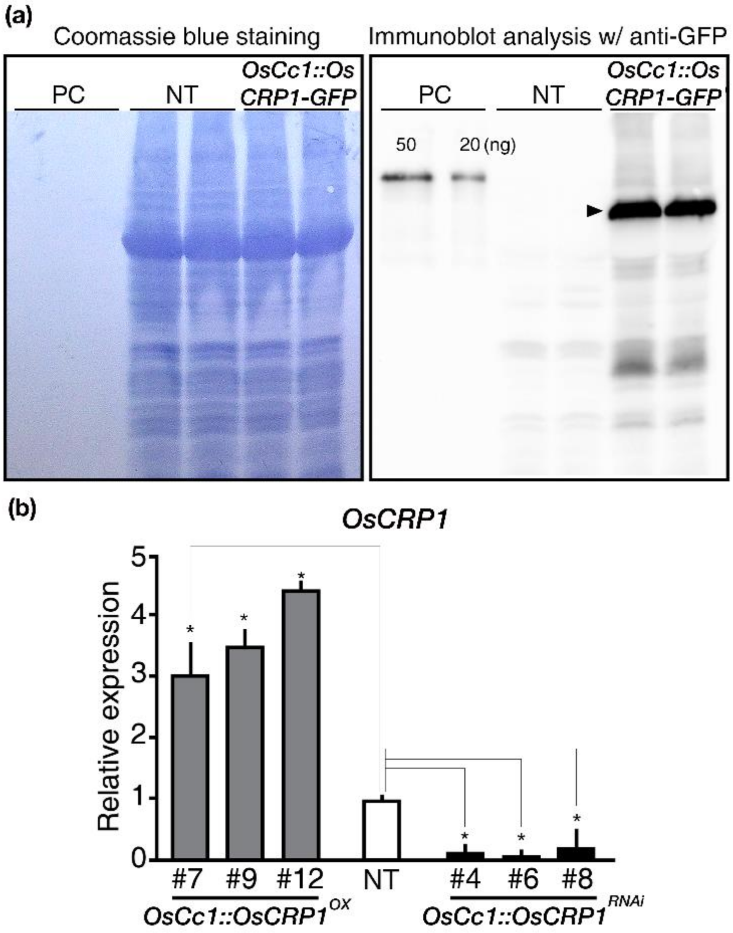

Figure S3. Protein and transcript levels in *OsCRP1* transgenic plants. A, Levels of OsCRP1-green fluorescent protein (GFP) fusion protein in *OsCc1::OsCRP1-GFP* plants as determined by immunoblot analysis using α-GFP antibody. Fifteen μg of total soluble protein was separated by SDS-PAGE in duplicates. Arrowhead indicates OsCRP1-GFP at the expected size (55 kDa). The protein loading is shown by CBB (Coomassie Brilliant Blue) staining in the lower panel. The PC (positive control) (PolyTag-GFP, Shanghai Genomics, China) is 74 kDa. B, Expression level of *OsCRP1* in lines chosen for further study. Total RNAs extracted from two-week-old rice leaves were used for qRT-PCR analysis. All the values were normalized to the internal *OsUbi1* control gene, and data bars represent the mean ± SD of two biological replicates, each of which had three technical replicates. Values for the non-transgenic (NT) samples were set as 1.0 for normalization.

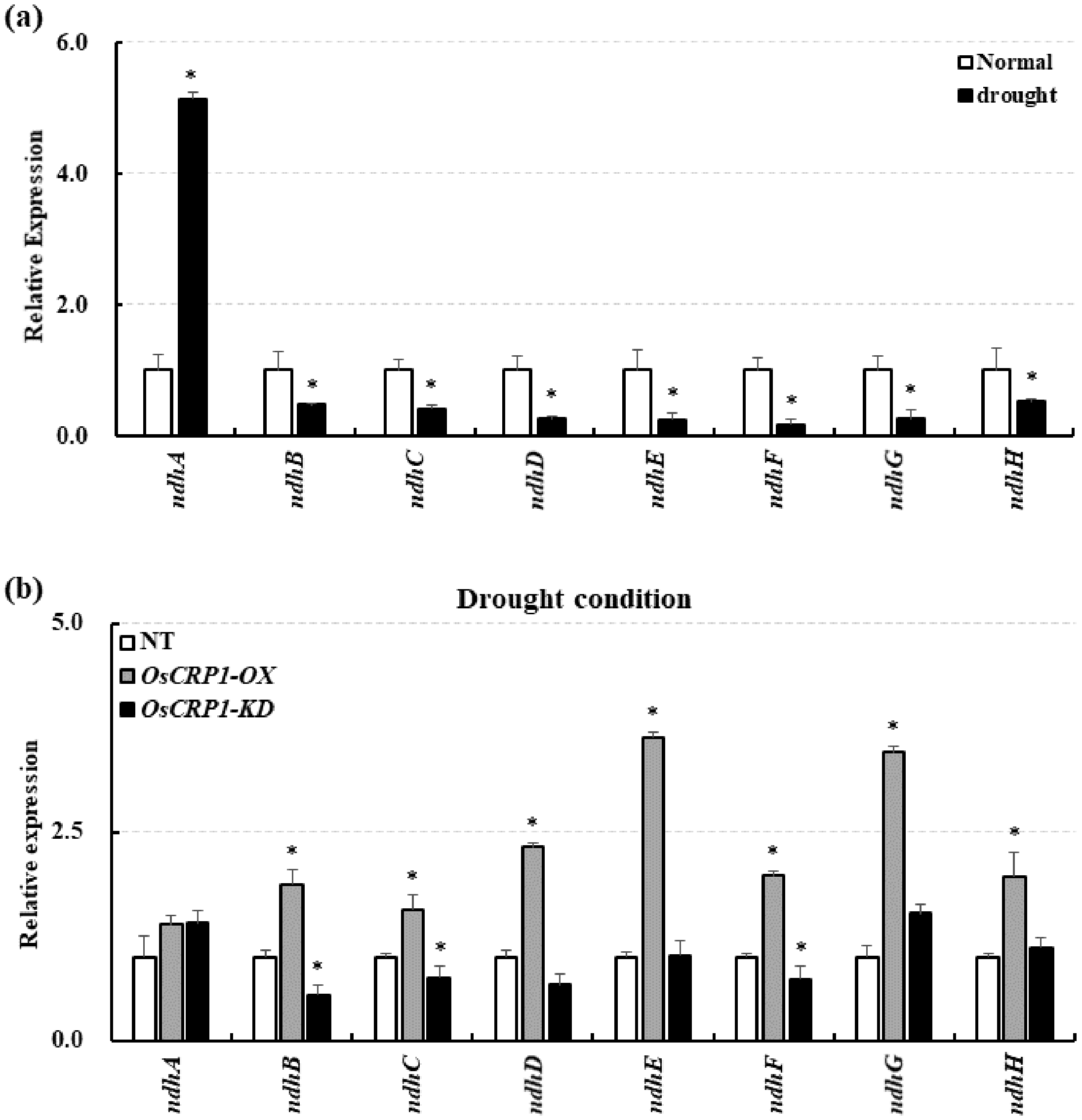

Figure S4. Relative expression levels of *ndh* genes after drought treatment. A, Transcription level of *ndh* genes in the non-transgenic (NT) plant after drought treatment (3h air-drying). Values for the Normal (without treatment) samples were set as 1.0 for normalization. Asterisks indicate significant differences compared with Normal ($P < 0.05$, t-test) B. Transcription level of *ndh* genes in NT and transgenic plants (*OsCRP1* overexpressing and knock-down) after drought treatment (3h air-drying). Total RNAs extracted from two-week-old rice leaves were used for qRT-PCR analysis. All the values were normalized to the internal *OsUbi1* control gene, and data bars represent the mean ± SD of three bio-logical replicates, each of which had three technical replicates. Values for the NT samples were set as 1.0 for normalization. Asterisks indicate significant differences compared with NT ($P < 0.05$, One-way ANOVA)

Table S1 List of primers used in this study.

Target Gene		Primer Sequence	
		Forward	Reverse
OsCRP1	Cloning	5'-TATACAGAGGAGACTCGATTGA-3'	5'-TACATACACAGCTGATGTTGAC-3'
gfp	Cloning	5'-CAGCACGACTTCTTCAAGTCC-3'	5'-CTTCAGCTCGATGCGGTTCAC-3'
OsCRP1	Localization	5'-TTGCTCCGTGGATCCTCGATGGCC TCC TCCATCGCCAT-3'	5'-AAAGCGGCCGCAAATAAAGCCTCG G CGCGGTGGTCGCTC-3'
OsUbi1	qRT-PCR	5'-ATGGAGCTGCTGCTGTTCTA-3'	5'-TTCTTCCATGCTGCTCTACC-3'
OsCRP1	qRT-PCR	5'-CAGCTGTTCAGCGAGCATGG-3'	5'-CGCTCCTCTGCAACATTCAC-3'
ndhA	qRT-PCR	5'-CGAGCTGCCGCTCAATCTAT-3'	5'-AGGCTGACGCCAAAGATTCC-3'
ndhB	qRT-PCR	5'-TACGAAGGATCCCCCACTCC-3'	5'-TCCAGAAGAAGATGCCATTCGT-3'
ndhC	qRT-PCR	5'-GCCCAAGGGTAGAGAAAGACC-3'	5'-TTAGCTCCGGTTCGTGAAGG-3'
ndhD	qRT-PCR	5'-GCTTCCCCATGGGTATCTGG-3'	5'-ACGGTTCCAACGAACCAAGA-3'
ndhE	qRT-PCR	5'-AGCCGCAAGGGCTATAACAA-3'	5'-TGATCACAAGCCGAAACATGG-3'
ndhF	qRT-PCR	5'-CGCAGTTTTTCGACAAGGGT-3'	5'-GACGAAATTCGACCTCCCCC-3'
ndhG	qRT-PCR	5'-GTTGTGCCACAGCTACAAAGT-3'	5'-GGGGGTCTAGGGGTGGTATT-3'
ndhH	qRT-PCR	5'-ATAAGGCAGATCGGCAGCAA-3'	5'-TTATGGCAGATCTCGGTGCG-3'
psaA	qRT-PCR	5'- AGTAGCAGTAGGCGGCAAAG -3'	5'- ACGGGAACTGCGAGCAAATA -3'
psaB	qRT-PCR	5'- TGGACATCTTGTTTGGGCGA -3'	5'- GAGCCACGGGCTTATCTCTC -3'
psaC	qRT-PCR	5'- AAGATAGAGCCATGCTGCGG -3'	5'- ACCGAAGATTGTGTGGGTTGT -3'
psbB	qRT-PCR	5'- ACACATTTGGCATGGGGCTA -3'	5'- ACTGGCTGTCTCCCTGTAGT -3'
psbC	qRT-PCR	5'- AATGTGGGATCTGCCCAAGG -3'	5'- CACGAAGGTCCCAAAAACGC -3'
psbD	qRT-PCR	5'- GGCAACCGTGGAAAACACTC -3'	5'- AAAGCGATTAGCGGTGACCA -3'
psbE	qRT-PCR	5'- ACGGAATTCCTTGTCGGCTT -3'	5'- CCCTATTCATTGCGGGTTGG -3'
psbF	qRT-PCR	5'- CGTTGGATGAACTGCATTGCT -3'	5'- TTTTACAGTGCGATGGCTGG -3'
psbG	qRT-PCR	5'- CCGCTTGCCTAGGACTTGAT -3'	5'- AGACTCTCTAGTTTATGGCCCCT -3'
psbH	qRT-PCR	5'- CCAAGACAAACTCGCGTAGG -3'	5'- CGCGAATAAAGCCATTGCGA -3'
psbK	qRT-PCR	5'- TTTCTTCGCCAAATTGCCCG -3'	5'- CAGCTTGCCAAACAAAGGCT -3'
atpA	qRT-PCR	5'- TGAATCTCCTGCTCCGGGTA -3'	5'- GCTGTTTTGCCGGTTTGTCT -3'
atpB	qRT-PCR	5'- CCTTATCGGCGTGGAGGAAA -3'	5'- CCCCTACTCCGCCAAATACG -3'
atpE	qRT-PCR	5'-TGTTAATGGGGGCGTGGTTT-3'	5'-TGACTCCTAAGCGAATTATTTGGG-3'
atpF	qRT-PCR	5'-CCGACAACGGGTTTTCCAAC-3'	5'-ATTCCATGGCCCCGAGAATG-3'

Table S2 List of chloroplast genes in *OsCRP1* overexpression and knock-down compared with non-transgenic plants based on the RNA-seq data

**Description**	OX/NT fold change	OX/NT p-values	RNAi/NT fold change	RNAi/NT p-values
psaA	0.91	0.518	0.73	0.030
psaB	0.96	0.746	0.81	0.244
psaC	0.60	0.006	0.78	0.239
psbB	1.09	0.400	1.08	0.581
psbC	1.00	0.990	1.01	0.956
psbD	1.18	0.345	0.90	0.510
psbE	0.84	0.574	1.34	0.393
psbF	0.84	0.459	1.23	0.433
psbG	1.07	0.251	0.98	0.238
psbH	1.30	0.426	0.86	0.651
psbK	0.39	0.112	0.29	0.045
atpA	1.60	0.036	0.51	0.022
atpB	4.33	0.055	1.52	0.265
atpE	2.70	0.106	0.36	0.193
atpF	1.10	0.411	0.64	0.021
ndhA	5.37	0.049	1.17	0.918
ndhB	2.16	0.039	0.61	0.050
ndhC	1.21	0.230	0.59	0.019
ndhD	1.68	0.298	0.65	0.036
ndhE	4.28	0.025	0.37	0.023
ndhF	1.50	0.179	0.13	0.029
ndhG	1.70	0.382	0.25	0.025
ndhH	1.90	0.196	0.91	0.903
